# Woman

**Published:** 1884-02

**Authors:** 


### WOMAN.

The admirable address of Dr. N. W. Kingsley, before the American Academy of Dental Science in Boston, will appear in full in the March Number of the INDEPENDENT PRACTITIONER.

There was not sufficient space in this number for the whole of it, and it would be an injustice to both the author and the reader to divide it.

